# Postoperative hungry bone syndrome in primary hyperparathyroidism: A case report

**DOI:** 10.1097/MD.0000000000039717

**Published:** 2024-09-20

**Authors:** Bin Li, XiaoXu Lv, XiaoMing Li, XiaoZhi Hou, FengLei Xu

**Affiliations:** a Department of Otorhinolaryngology, Head and Neck Surgery, Shandong Provincial Hospital Affiliated to Shandong First Medical University, Jinan, Shandong, China.

**Keywords:** bone turnover, HBS, iPTH, PTX, PHPT

## Abstract

**Rationale:**

Hungry bone syndrome (HBS) is a forgotten and underdiagnosed cause. Postoperative HBS refers to patients with high bone turnover before surgery, but after surgery, the inhibition of osteoclast resorption by intact parathyroid hormone suddenly decreases, resulting in a sudden increase in the amount of calcium resorbed by the bone, and a rapid, severe and persistent hypocalcemia, which may be accompanied by hypophosphatemia and hypomagnesemia. We present a case with information about HBS and related complications after parathyroidectomy (PTX).

**Patient concerns:**

The patient was a 57-year-old woman who presented to the hospital with “pain in both ankles for more than 3 years and in both knees for more than 2 years.”

**Diagnoses:**

A parathyroid mass was found preoperative. Unilateral resection of the lesion was performed under general anesthesia. On gross examination, the mass was reddish brown in color, about 2.9 × 2.5 × 2.3 cm, with abundant blood supply. Postoperative pathology diagnosed parathyroid adenoma.

**Interventions:**

The patient was diagnosed with HBS on day 3 post-PTX, which lasted for 9 days.

**Outcomes:**

After active calcium supplementation and other pharmacological interventions, her test parameters gradually returned to normal and she was discharged on the 13th day after surgery.

**Lessons:**

Using the case of a patient with primary hyperparathyroidism with HBS lasting 9 days after PTX for diagnosis and management, we aimed to summarize possible predictors and perioperative management strategies to reduce the incidence, severity, and duration of postoperative HBS.

## 1. Introduction

Rapid, severe and persistent hyponatremia and its range of complications following parathyroidectomy (PTX) are referred to as postoperative hungry bone syndrome (HBS) in the literature.^[1]^ HBS refers to patients with high bone turnover before surgery, but after surgery, the inhibition of osteoclast resorption by intact parathyroid hormone (iPTH) suddenly decreases, resulting in a sudden increase in the amount of calcium resorbed by the bone, and a rapid, severe and persistent hypocalcemia, which may be accompanied by hypophosphatemia and hypomagnesemia.^[2]^ This occurs most frequently in patients with severe primary hyperparathyroidism (PHPT), secondary hyperparathyroidism, or tertiary hyperparathyroidism following PTX therapy. We report a case of HBS with the aim of illustrating the pathogenesis, pathophysiology, and perioperative treatment strategies behind this type of disease.

## 2. Case presentation

**Figure 1.**
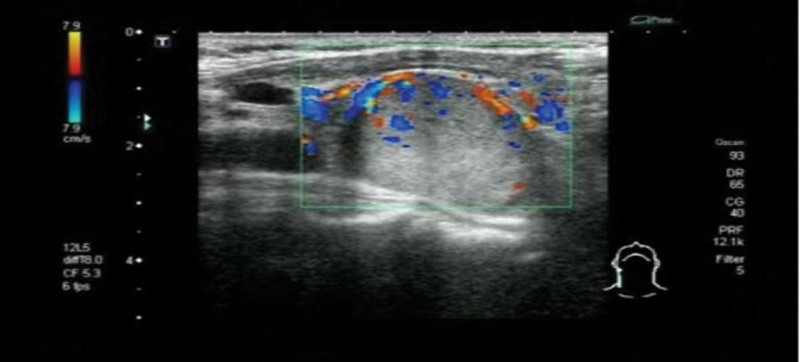
Substantial nodule in the dorsal aspect of the right middle lobe of the thyroid gland.

**Figure 2.**
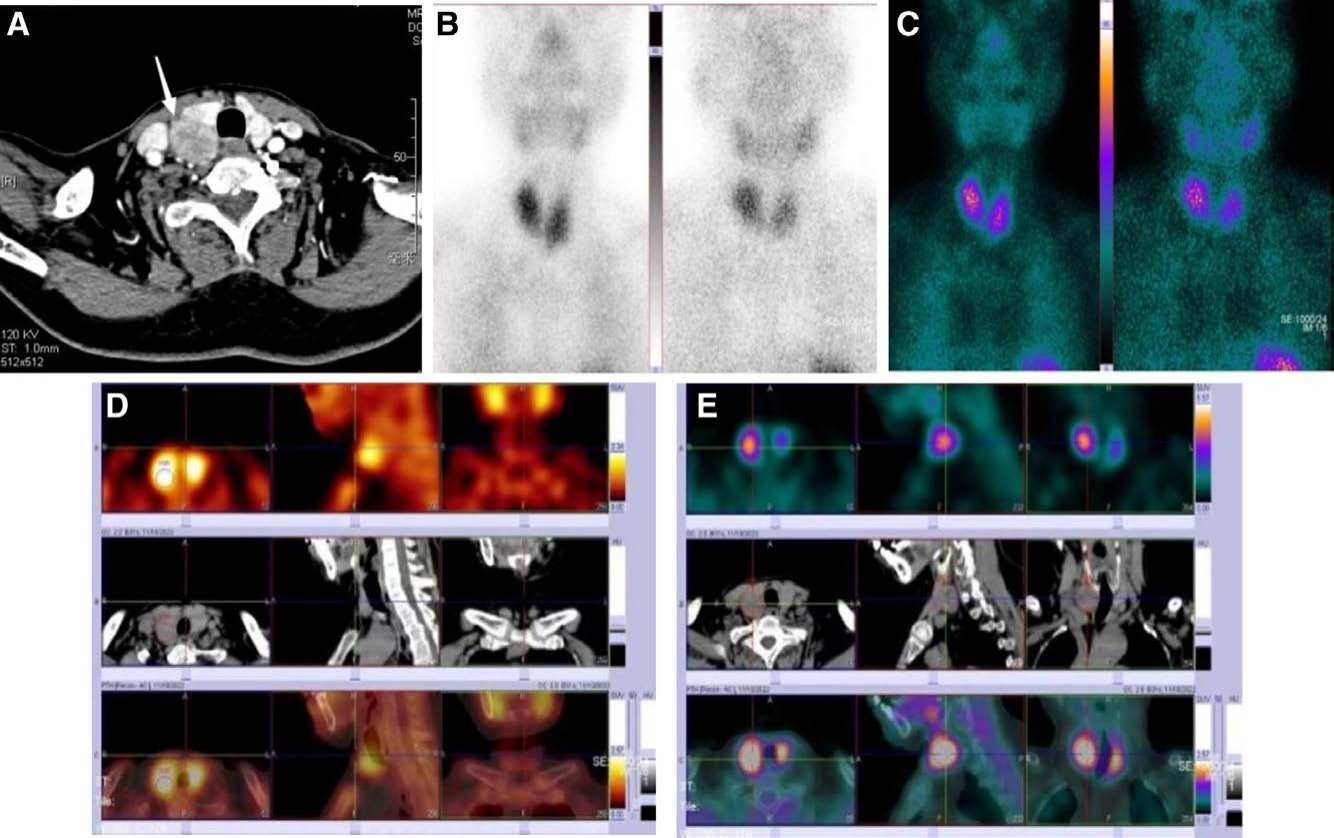
(A) Arrow pointing to foci of rounded soft tissue density in the right lobe of the thyroid gland. (B) Abnormal radiation from the right lobe of the thyroid gland. (C) Radiologically limited abnormal increase in the right lobe of the thyroid gland. (D) Hypodensity of a dorsal roundish mass in the right lobe of the thyroid gland. (E) The posterior border of the thyroid gland is well demarcated and radioactivity uptake is markedly increased.

**Figure 3.**
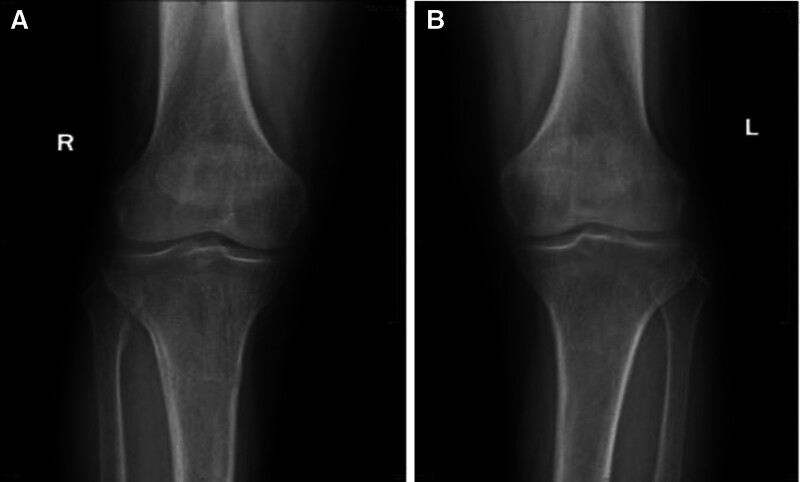
(A) Decreased bone density in the right knee. (B) Decreased bone density in the left knee.

A 57-year-old female patient was admitted with “pain in both ankles for more than 3 years and in both knees for more than 2 years,” accompanied by weakness of the limbs, depression (moaning), gastrointestinal discomfort, poor diet and sleep. Blood chemistry on day 1 of admission: parathyroid hormone (PTH) 3203↑(15–65) pg/mL, alkaline phosphatase (ALP) 1926↑(23–140) U/L, serum calcium 3.18↑(2.2–2.7) mmol/L, serum phosphorus 0.71↓(0.83–1.48) mmol/L, serum magnesium 0.73↓(0.75–1.02) mmol/L, thyroglobulin 127.80↑(3.5–77) ng/mL, urine protein ±15 mg/dL↑. Ultrasonography (Fig. 1): an isoechoic nodule was detected in the dorsal parenchyma of the right middle lobe of the thyroid gland, about 2.9 × 2.3 × 2.3 cm, with clear boundaries, regular morphology, and homogeneous internal echogenicity; color doppler flow imaging: striated blood flow signals were detected inside the nodule, and the origin of the parathyroid glands was considered. Enhanced computed tomography (CT) (Fig. 2A): the right lobe of the thyroid gland was posterior to the rounded soft tissue density foci, with uneven density and speckled low-density areas, smooth edges and clear borders, and the foci strengthened markedly after enhancement, and the demarcation between the foci and the thyroid gland and the surrounding tissues was still clear; the right lobe of the thyroid gland was slightly shifted forward, and the cervical and thoracic vertebral bone in the scanning field was hypodense, with sparse trabeculae; emission computed tomography 99mTc-MIBI dual-phase imaging showed limited abnormal radioactivity in the right lobe (Fig. 2B). Emission computed tomography 99mTc-MIBI dual-phase imaging showed that the right lobe showed limited abnormal increase in radioactivity (Fig. 2C); single-photon emission computed tomography/CT: the dorsal right lobe showed a rounded mass with decreased density (Fig. 2D), clear border, clear demarcation from the posterior border of the thyroid gland, and obvious increase in radioactivity uptake (Fig. 2E), so that parathyroid lesions were considered. Bilateral knee joints in front and side view showed slightly reduced bone density (Fig. 3A and B); dual-energy X-ray bone densitometry showed that “the average bone density of lumbar vertebrae L1 to L4 (−5.9 SD) (range: −1.0 to 1.0 SD); the average bone density of the right hip (−4.4 SD) (range: −1.0 to 1.0 SD); the average bone density of the left hip (−4.6 SD); the average bone density of the left hip (−4.6 SD) (range: −1.6 SD); and the average bone density of the left hip (−4.6 SD) (range: −1.0 to 1.0 SD), and osteoporosis was considered”. Preoperative diagnosis: (1) PHPT (right parathyroid lesion); (2) secondary hypercalcemia; (3) secondary hypophosphatemia and hypomagnesemia; and (4) secondary osteoporosis.

**Figure 4.**
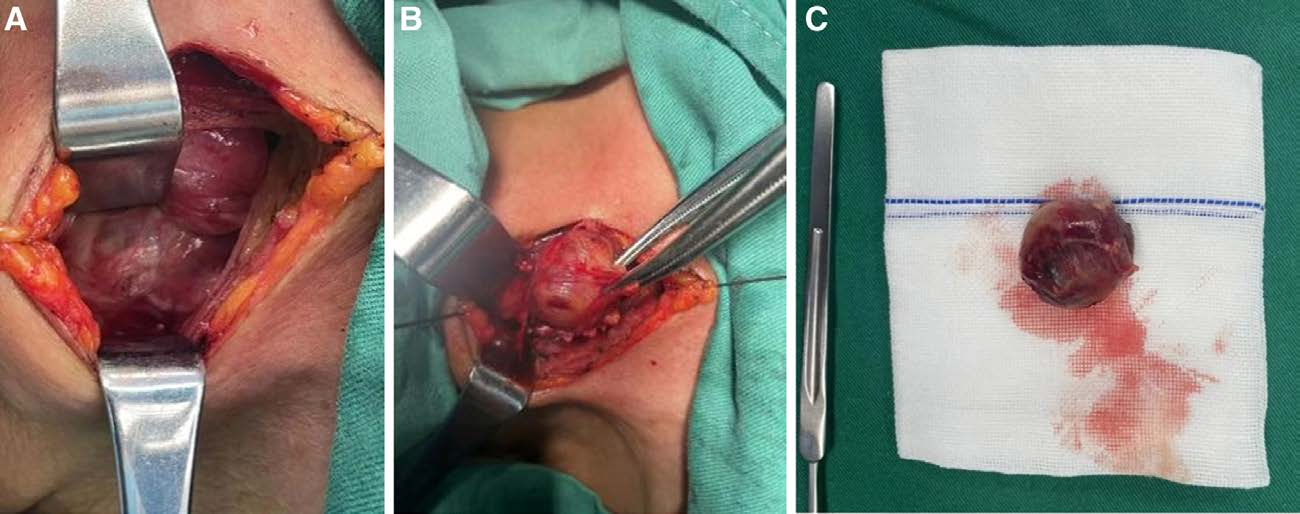
(A) Exposure of the right lower pole of the thyroid gland and separation of the surrounding fatty envelope. (B) Markedly enlarged adenomatous lesion seen in the right inferior parathyroid region. (C) Individual glands removed.

**Figure 5.**
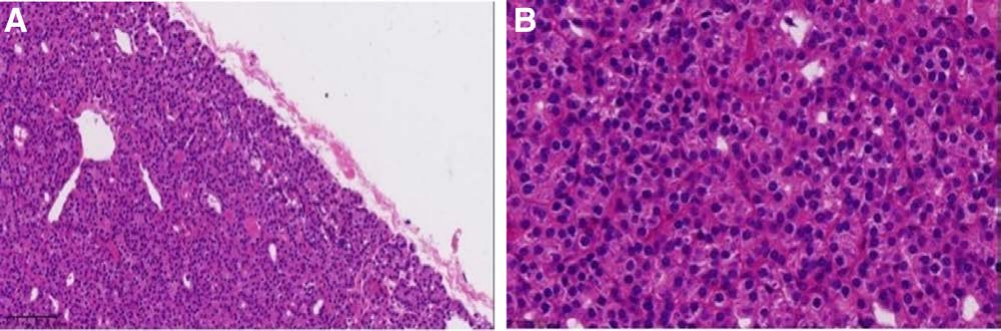
(A) Postoperative pathology (HE × 100). (B) Postoperative pathology (HE × 40). HE = hematoxylin-eosinstaining.

On day 6 since admission, the right parathyroid lesion was resected and the central zone lymph nodes were explored under general anesthesia with endotracheal intubation and intraoperative parathyroid hormone monitoring, and a 3 to 4 cm long curved incision was made along the dermatomal line of the neck in the low position, and the side of the lesion was explored according to the information of the preoperative image localization. The right inferior pole of the thyroid gland was fully exposed and lifted up, and the surrounding fat capsule and peritoneum were separated (Fig. 4A), and the recurrent laryngeal nerve was protected, and a clearly enlarged adenomatous lesion was seen in the right inferior parathyroid region (Fig. 4B). After complete resection of the lesion, the intraoperative PTH plasma levels (iPTH) decreased to 1493 pg/mL, 520 pg/mL, and 263 pg/mL at 0, 10, and 30 minutes, respectively, and the exploration of the remaining glands was terminated. The resected single gland was reddish-brown in color, rich in blood supply, about 2.9 × 2.5 × 2.3 cm, and weighed about 10 g (Fig. 4C). Postoperative pathology was parathyroid adenoma (Fig. 5A and B); immunohistochemical analysis was PTH(+), thyroglobulin(−), thyroid transcription factor-1(−), ki-67(3%), thyroid peroxidase(+), cytokeratin-19(+), galectin-3(−).

**Figure 6.**
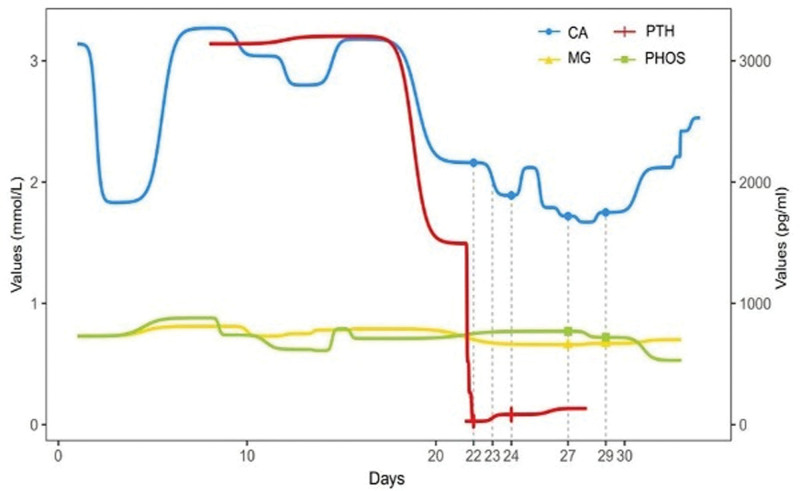
Graph of changes in CA, PTH, MG, and PHOS during the patient’s hospitalization. CA = calcium, MG = magnesium, PHOS = phosphorus, PTH = parathyroid hormone. (The values corresponding to the horizontal coordinates are the dates after the patient’s admission to the hospital, and the values of the horizontal coordinates corresponding to the dashed lines in the graph are the dates of the pharmacological interventions, which are postoperative day 1, postoperative day 2, postoperative day 3, postoperative day 6, and postoperative day 8, respectively).

Changes in calcium, PTH, magnesium, and phosphorus during the hospitalization period were as follows (Table 1). The lab values were monitored daily, as shown in the table, and that they were adjusted accordingly. On postoperative day 1, the patient was given calcium. On postoperative day 2, because of numbness in the face, hands and feet and pain in the ribs, oral calcium supplementation and calcium chloride were given. On postoperative day 3, the patient was given intravenous calcium chloride, oral magnesium was added on postoperative day 6, and intravenous calcium gluconate was given on postoperative day 8 (Fig. 6).

**Table 1:** The significant laboratory test results during hospitalization.

Dates	Lab values			
	CA (2.2–2.7) mmol/L	MG (0.75–1.02) mmol/L	PTH (15–65) pg/mL	PHOS (0.83–1.48) mmol/L
Day 1 of admission	3.18	0.73	3203	0.71
Postoperative day 1	2.16	–	27.64	–
Postoperative day 3	1.89	–	83.57	–
Postoperative day 4	2.12	–	–	–
Postoperative day 5	1.79	–	–	–
Postoperative day 6	1.72	0.66	–	0.77
Postoperative day 7	1.67	–	133.00	–
Postoperative day 8	1.75	0.67	–	0.72
Postoperative day 9	1.83	–	–	–
Postoperative day 11	2.21	–	–	–
Postoperative day 12	2.42	0.70	–	0.53
Postoperative day 13	2.53	–	–	–
3 months follow-up	2.10	0.83	60	1.45

CA = calcium, MG = magnesium, PHOS = phosphorus, PTH = parathyroid hormone.

## 3. Discussion

The prevalence of PHPT is about 0.86%, most commonly seen in postmenopausal women, with a male-to-female incidence of 1:3 to 1:4.^[1]^ Overproduction of PTH by 1 or more diseased parathyroid tissues leads to hypercalcemia, hypophosphatemia, kidney stones and increased bone resorption mainly in cortical bone.^[2]^ PHPT includes asymptomatic, symptomatic and normocalcemic forms, and the guidelines for the treatment of asymptomatic PHPT are as follows serum calcium (above normal) of 1.0 mg/dL; creatinine clearance < 60 cc/min, 24-hour urinary calcium > 400 mg, high stone risk; *T*-score ≤ −2.5 (any location) on dual-energy X-ray bone densitometry bone density scanning at 3 sites (lumbar spine, total hip, femoral neck, or distal 3rd of radius), fragility fracture, and vertebral compression fracture on spinal imaging.^[3]^ Compression fracture,^[3]^ symptomatic PHPT can involve multiple systems such as bone, kidney, gastrointestinal tract and nerves, etc. Intraoperative parathyroid hormone monitoring includes Vienna criterion, Halle criterion, Miami criterion, Rome criterion, and the commonly used “Vienna criterion.” The “Vienna criterion” is a ≥ 50% decrease in iPTH within 10 minutes after resection from the pre-skinning “baseline.”^[4]^ The majority of PHPTs are monoadenomatous lesions (75–85%), followed by multiple lesions (hyperplasia in 10–15%; double adenomas in 2–12%) and carcinoma (1%).^[5]^

HBS is defined as a condition of albumin-corrected serum calcium ≤ 2.1 mmol/L and normal or elevated serum whole iPTH levels, which develops on or after the 3rd postoperative day or persists for more than 3 days after surgery. The prevalence is about 2.4% after PTX in PHPT patients with high bone turnover.^[6]^ Fuller Albright was the first to describe PHPT patients with “stones, bones, and groans” in 1930, and the first to mention “HPT and groans” in a patient with PHPT, and was also the first to mention HBS.^[7]^ In our case, the presence of serum calcium ≤2.1 mmol/L on the 3rd postoperative day and the persistent elevation of serum iPTH for more than 3 days were consistent with the diagnosis of postoperative HBS after PTX in a patient with symptomatic PHPT. Preoperative prediction of HBS risk factors is particularly important: (1) some scholars believe that preoperative PTH > 409 pg/mL can predict HBS^[8]^; (2) because menopausal women due to the reduction of estrogen accelerated bone loss, easy to combine with malnutrition, vitamin D deficiency, some scholars believe that postmenopausal women of advanced age is a predictor of HBS^[9]^; (3) removal of the parathyroid lesion volume or weight can be used as a predictor^[10]^; (4) the volume or weight of parathyroid lesions can be a predictive indicator^[10]^; (5) the volume or weight of parathyroid lesions can be a predictive indicator^[10]^; (6) the volume or weight of parathyroid lesions can be a predictive indicator.^[10]^ In the study of Ko et al,^[11]^ ALP mainly reflects bone resorption or destruction in bone metabolism, so preoperative ALP is a predictor of HBS, and preoperative increase in osteocalcin is also an independent risk factor; some scholars^[12,13]^ believe that serum 25-hydroxyvitamin D is sufficient, insufficient, deficient, and severely deficient in the following criteria: 30 to 100 ng/mL, 20 to 30 ng/mL, <20 to 20 ng/mL, and < 30 ng/mL, respectively; the criteria for adequate, inadequate, deficient, and severely deficient serum 25-hydroxyvitamin D are 30 to 100 ng/mL, 20 to 30 ng/mL, and < 20 to 90 ng/mL, respectively. 30 ng/mL, <20 ng/mL, <10 ng/mL, and severe preoperative vitamin D deficiency is a risk factor; Paepegaey et al^[14]^ suggested that preoperative 18F-fluorocholine positron emission tomography/CT suggestive of intense diffuse bone uptake may be a predictor of HBS in patients with PHPT. In addition bone mineral density, testosterone, 24 hours urinary calcium and albumin are also associated with HBS.

There is often not enough awareness of HBS. The most common cause of postoperative hypocalcemia after PTX is impaired parathyroid blood supply characterized by low PTH, whereas HBS characterized by elevated PTH and increased bone formation is not so common. The sharp decrease of iPTH release after PTX, the sudden decrease of inhibition of osteoclast resorption, and the sudden decrease of osteoclast function broke the unstable equilibrium between large amount of bone calcium outflow and blood calcium inflow in the preoperative abnormally high osteoconversion pathophysiological state, and osteogenesis was greater than osteogenesis, and a large amount of blood calcium and phosphorus entered into the bone, and blood calcium and phosphorus of bone resorption were suddenly increased, and the frequency of the activation of the new bone reconstruction sites was decreased, and the bone reconstruction intervals were activated. The frequency of new bone remodeling sites decreases, and the bone remodeling gap decreases, leading to a rapid, severe or even continuous decline in serum calcium, phosphorus and magnesium, manifesting electrolyte disorders such as secondary hypocalcemia, hypophosphatemia and hypomagnesemia.^[15]^ Secondary hypocalcemia is associated with a secondary elevation of PTH, and hypophosphatemia is more common in postoperative patients with decreased bone resorption and increased bone formation and normal urinary phosphorus excretion. Hypomagnesemia promotes refractory hypocalcemia by decreasing iPTH secretion and inducing iPTH resistance. This theory is supported by Liu et al^[16]^ who found a rapid decrease in calcium and phosphorus levels and an increase in the number of mineralized nodules in cultures of osteoblast cell lines cultured in vitro at persistently high PTH concentrations. The short-term goal of HBS treatment is to replenish the circulating calcium deficiency and depleted skeletal calcium reserves, while the long-term goal is to normalize osteoconversion and remineralization of bone, and to try to normalize osteoconversion with active calcium supplementation postoperatively, and to try to recombine PTH, if necessary. The long-term goal is to normalize bone turnover and bone remineralization, with aggressive postoperative calcium supplementation and, if necessary, recombinant PTH.^[17]^

Hungry bone syndrome can also occur in other high bone conversion states after non-PTX, such as after total thyroidectomy for hyperthyroidism and in some patients with bone metastases from malignant tumors. In 2019, Kusuki and Mizuno^[18]^ reported a case of post-thyroidectomy HBS in a patient with Graves’ disease with severe thyrotoxicosis, elevated serum alkaline phosphatase, and low bone mineral density. Postoperatively, N-terminal prepeptide of pre-collagen type 1 increased significantly and anti-tartrate acid phosphatase 5b decreased indicating that post-thyroidectomy HBS persisted for 4 months. increase and decrease in anti-tartrate acid phosphatase 5b indicated that bone formation exceeded bone resorption after thyroid surgery and postoperative HBS lasted for 4 months. In 2018, Karunakaran et al^[19]^ reported 39% incidence of HBS after total thyroidectomy in hyperthyroidism patients. In 2011, Huang et al^[20]^ reported 2 cases of post-thyroid surgery HBS in hyperthyroidism patients, and suggested that thyroid hormone hyperactivation of osteoclast thyroid hormone receptor in hyperthyroidism was another uncommon cause of post-thyroid surgery HBS, and suggested that low magnesium in the post-thyroid surgery period should be the direct basis of diagnosis of HBS. In 2020 and 2018, there were case reports of HBS in patients with bone metastasis of prostate cancer and bone metastasis of gastric cancer.^[21,22]^ In 1997, see^[23]^ found that the incidence of hypocalcemia unrelated to hypothyroidism after subtotal thyroidectomy in patients with hyperthyroidism was 53%.

In conclusion, HBS may occur not only after PTX, but also after thyroidectomy for hyperthyroidism and after other abrupt changes in pathophysiologic status of high bone conversion. Risk factors for the development of HBS include age, weight/volume of parathyroid glands removed, imaging evidence of bone disease, and vitamin D deficiency. Therefore, it is important to pay close attention to ALP, iPTH, 24-hour urinary calcium, urinary creatinine, and the presence of vitamin D deficiency, as well as abnormalities in bone resorption and bone formation markers, and to aggressively supplement with calcium and vitamin D. The use of preoperative bisphosphonates and bone resorption antagonists mitigates the hyperosteoconversion. The pathophysiological state of high bone conversion can be alleviated by using bisphosphonates and bone resorption antagonists preoperatively, and the surgery can even be appropriately delayed if necessary; postoperatively, calcium supplementation should be actively used, and restructuring of PTH should be attempted if necessary, so as to restore bone conversion to normal, thereby reducing the incidence and severity of postoperative HBS.

## Author contributions

**Investigation:** Xiaoxu Lv, Xiaoming Li, Xiaozhi Hou.

**Writing – original draft:** Bin Li.

**Writing – review & editing:** Fenglei Xu.
